# Comparison of the Deep Optic Nerve Head Structure between Normal-Tension Glaucoma and Nonarteritic Anterior Ischemic Optic Neuropathy

**DOI:** 10.1371/journal.pone.0150242

**Published:** 2016-04-01

**Authors:** Eun Ji Lee, Yun Jeong Choi, Tae-Woo Kim, Jeong-Min Hwang

**Affiliations:** Department of Ophthalmology, Seoul National University College of Medicine, Seoul National University Bundang Hospital, Seongnam, Korea; University of Iowa, UNITED STATES

## Abstract

**Purpose:**

To compare the deep optic nerve head (ONH) structure between normal-tension glaucoma (NTG) and nonarteritic anterior ischemic optic neuropathy (NAION) and also in healthy subjects as a control using enhanced depth imaging (EDI) spectral-domain optical coherence tomography (SD-OCT).

**Methods:**

This prospective cross-sectional study included 21 NAION patients who had been diagnosed as NAION at least 6 months prior to study entry, and 42 NTG patients and 42 healthy controls who were matched with NAION patients in terms of age, intraocular pressure (IOP), and optic disc area. The retinal nerve fiber layer (RNFL) thickness in the affected sector was also matched between NAION and NTG patients. The ONH was imaged using SD-OCT with the EDI technique. The anterior lamina cribrosa surface depth (LCD) and average prelaminar tissue (PT) thickness were measured in a sector of interest in each eye and compared among the three groups.

**Results:**

In the sector-matched comparison, LCD was largest in NTG patients, followed by NAION patients, while PT was thinner in NTG patients than in NAION patients (all *P* < 0.001). NAION patients had a comparable LCD and a thinner PT relative to normal controls (*P* = 0.170 and < 0.001, respectively).

**Conclusion:**

The deep ONH configuration is strikingly different between NTG and NAION. The differing features provide comparative insight into the pathophysiology of the two diseases, and may be useful for differential diagnosis.

## Introduction

Both glaucoma and nonarteritic anterior ischemic optic neuropathy (NAION) involve loss of the retinal nerve fiber layer (RNFL). However, the topography of the optic disc is known to differ between these two entities. While excavation (i.e., cupping) of the optic nerve head (ONH) is a characteristic finding in glaucoma, no or only shallow cupping is observed in NAION [1]. It has been proposed that the difference in the disc topography between glaucoma and NAION is attributable to greater posterior displacement and/or thinning of the lamina cribrosa (LC) in glaucoma [2]. However, no previous study has compared the configuration of the deep optic nerve tissue including the LC between these two diseases.

Measuring the RNFL thickness is an important diagnostic tool in glaucoma. However, RNFL thinning is not specific to glaucomatous optic neuropathy (GON), which has led to the RNFL defects found in non-GON often being confused with glaucoma [2–7]. It has been demonstrated that the poor correlation between the neuroretinal rim area and the RNFL thickness may be useful for differentially diagnosing NAION from normal-tension glaucoma (NTG) [8]. We hypothesized that the configuration of the deep optic nerve tissue may provide another diagnostic clue for differentiating between NTG and NAION. In addition, comparing the deep optic nerve tissue may provide further insight into the pathophysiology of the two disease entities. Therefore, we performed this study to compare the deep optic nerve tissue configuration between NTG and NAION and also in healthy controls using enhanced depth imaging (EDI) spectral-domain optical coherence tomography (SD-OCT).

## Methods

### Study Subjects

This study involved NAION patients who visited Seoul National University Bundang Hospital (SNUBH) from January 2012 to September 2014. The study was approved by the SNUBH Institutional Review Board and conformed to the Declaration of Helsinki. Written informed consent was obtained from all patients.

All of the patients underwent a complete ophthalmic examination including visual acuity (VA) assessment, refraction test, slit-lamp biomicroscopy, gonioscopy, Goldmann applanation tonometry, and dilated stereoscopic examination of the optic disc. They also underwent stereo disc photography, circumpapillary RNFL thickness measurement and EDI optic disc scanning using SD-OCT (Spectralis OCT, Heidelberg Engineering, Heidelberg, Germany), standard automated perimetry (Humphrey Field Analyzer II 750; 24–2 Swedish interactive threshold algorithm; Carl-Zeiss Meditec, Dublin, CA, USA), and measurements of corneal curvature (KR-1800, Topcon, Tokyo, Japan), central corneal thickness (Orbscan II, Bausch & Lomb Surgical, Rochester, NY, USA), and axial length (IOL Master version 5, Carl-Zeiss Meditec).

To be included in the study, subjects were required to be diagnosed as NAION at least 6 months prior to study entry. The NAION diagnosis was made based on the sudden, painless loss of VA without history of glaucoma or retinal diseases, optic disc edema with or without superficial hemorrhages at the optic disc border and adjacent retina on fundus ophthalmoscopy, or visual field (VF) defects consistent with NAION, and with spontaneous resolution of optic disc edema within 2 to 3 months. The exclusion criteria were a symptom or sign suggesting arteritic ischemic optic neuropathy or giant cell arteritis such as jaw claudication, anorexia, unattended weight loss, and elevated erythrocyte sedimentation rate or reactive protein C levels.

NTG patients were selected from our database that was assembled for the Lamina Cribrosa Exploration Study and the Investigating Glaucoma Progression Study. NTG was diagnosed based on the presence of glaucomatous optic nerve damage (i.e., notching, neuroretinal rim thinning, and RNFL defect) with corresponding VF loss, an open angle on gonioscopy, and an intraocular pressure (IOP) of ≤21 mmHg over multiple measurements during office hours (9 a.m. to 5 p.m.). A glaucomatous VF defect was defined as (1) outside normal limits on the glaucoma hemifield test; or (2) three abnormal points with a probability of *P*<5% of being normal, one with *P*<1% by pattern deviation; or (3) a pattern standard deviation with *P*<5% if the VF was otherwise normal, as confirmed in two consecutive tests. The VF results were deemed reliable when fixation losses were <20% and the false-positive and false-negative rates were both <25%.

Healthy eyes were defined as those with no history of ocular symptoms, disease, or intraocular surgery. Normal eyes also had an IOP of ≤21 mmHg, an absence of glaucomatous optic disc appearance, and no VF defect.

NTG patients and healthy controls were selected by matching them with NAION patients in terms of age, IOP, optic disc area (ODA), and RNFL thickness in the affected sector (in cases of NTG, see “Measurement of anterior lamina cribrosa surface depth and average prelaminar tissue thickness”). The specific parameter values used to indicate a match were as follows: age within 5 years (most patients were matched within 2 years), IOP within 1 mmHg, ODA within 0.1 mm^2^, and RNFL thickness within 10 μm. Each NAION patient was matched with two NTG patients and two healthy controls (i.e., 1:2:2 matching). ODA was measured by two experienced observers (E.J.L. and Y.J.C.) who were masked to patient clinical information using the built-in manual caliper tool designed to calculate the area of fundus images in Heidelberg Eye Explorer (version 1.7.1.0, Heidelberg Engineering), which is a viewer program provided with the Spectralis OCT device. The average of the two measurements (one made by each observer) was defined as the final ODA.

The following exclusion criteria were applied for the eyes: spherical refraction of less than –6.0 or more than +3.0 diopters and a cylinder correction of less than –3.0 diopters or more than +3.0 diopters, or having undergone previous intraocular surgery or having coexisting retinal disease (e.g., proliferative diabetic retinopathy or retinal vessel occlusion) or neurological disease (e.g., pituitary tumor) that could have affected the VF or caused sudden visual loss.

### Enhanced-Depth-Imaging Spectral-Domain Optical Coherence Tomography of the Optic Disc

The ONH was imaged using the EDI technique. The details and advantage of this technology for evaluating the deep ONH structure have been described previously [9–11].

The ONHs of the patients were imaged through undilated pupils using a 10×15-degree rectangle covering the optic disc. This rectangle was scanned with approximately 65 horizontal raster B-scan sections separated by 30–34 μm (the scan-line distance is determined automatically by the instrument). Each section had approximately 42 OCT frames averaged, which provided the best trade-off between image quality and patient cooperation [10]. Twenty-four radial scans centered on the optic disc were then also obtained.

Only eyes where acceptable scans (i.e., quality score > 15) were obtained from the sector of interest (see “Measurement of anterior lamina cribrosa surface depth and average prelaminar tissue thickness”) that allowed clear delineation of anterior border of the LC were included in the subsequent analysis. The keratometry value was entered into the OCT device before image acquisition to correct the magnification error when making transverse measurements (Spectralis User manual software version 5.6, page 81).

All EDI SD-OCT examinations of NAION subjects were conducted at least 6 months after the acute onset of the disorder, when the optic disc edema had resolved. Images of NTG eyes were obtained before starting any IOP-lowering treatment.

### Measurement of Anterior Lamina Cribrosa Surface Depth and Average Prelaminar Tissue Thickness

**Fig 1:**
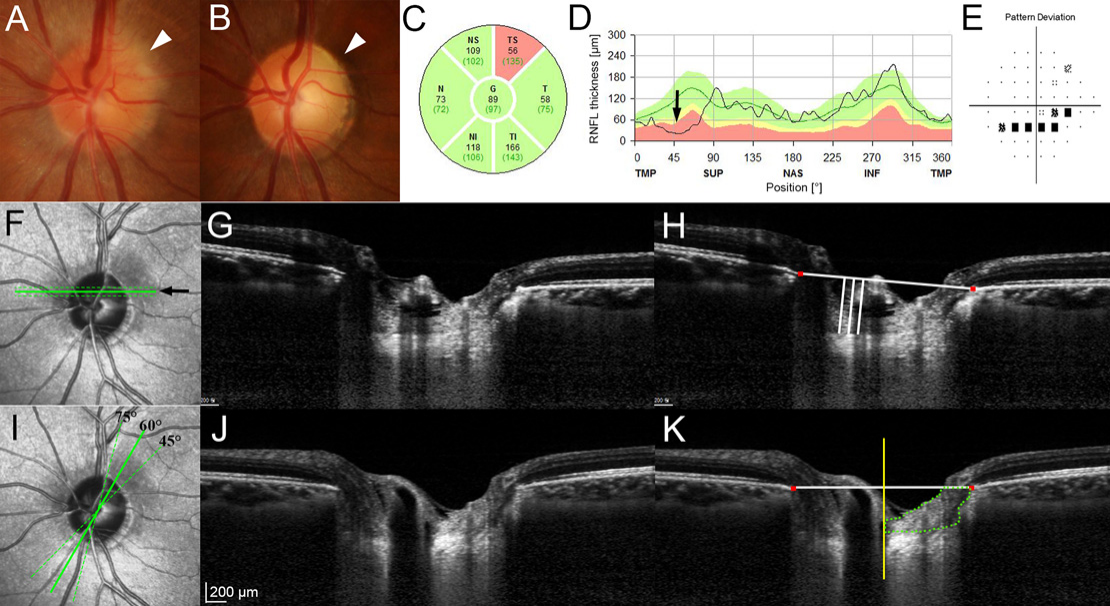
Measurement of the anterior lamina cribrosa surface depth (LCD) and the prelaminar tissue (PT) thickness (PTT) in the sector of interest. **(A)** Disc photograph obtained at the onset of nonarteritic anterior ischemic optic neuropathy (NAION). Note the blurred disc margin in the superotemporal sector (*arrowhead*). **(B)** Pallor is evident at the superotemporal neuroretinal rim 6 months later (*arrowhead*). Circular diagram **(C)** and TSNIT (temporal–superior–nasal–inferior–temporal) retinal nerve fiber layer (RNFL) thickness profile **(D)** at the 6-month follow-up. The superotemporal RNFL is thinner (*arrow*). **(E)** Inferior arcuate scotoma corresponding to the RNFL loss is evident. **(F)** Location of the three equidistant horizontal B-scans obtained from the middle third of the affected hemi optic nerve head (*solid and dotted lines*). **(G)** Horizontal B-scan image obtained from the location indicated by the *arrow* in the image on the left (F; *solid line*). **(H)** Same image shown in the image on the left (G) with labels. The LCD was measured as the perpendicular distance from the horizontal reference line (*horizontal line*) connecting the two terminations of Bruch’s membrane opening (BMO) (*red glyphs*) to the anterior laminar surface at three points: the maximally depressed point and two additional points that were 100 and 200 μm from the maximally depressed point in the temporal direction (*vertical lines*). **(I)** Location where the PTT was measured on three radial B-scans obtained from the affected sector (*solid and dotted lines*). **(J)** Radial B-scan image obtained from the 60-degree meridian shown in the image on the left (I; *solid line*). **(K)** The same image shown in the image on the left (J) with labeling. The PTT was measured by delineating the area surrounded by the vertical reference line (*yellow line*) from the center of BMO, anterior LC border, border tissue of Elschnig on the temporal side, the horizontal reference line connecting each BMO at the neuroretinal rim, and the internal limiting membrane (the area demarcated by *dotted line*). The average PTT was calculated by dividing the PT area by half of the BMO distance.

In NAION patients, either the superotemporal or inferotemporal sector that was affected by the disease was selected as the sector of interest (Fig 1). When both the superotemporal and inferotemporal sectors were affected, the more severely affected sector was selected based on the RNFL thickness. The NTG patients were matched with NAION patients based on the RNFL thickness in the sector of interest. The sector of interest in healthy controls was the sector of interest in the matched NAION patients.

The anterior LC surface depth (LCD) and the prelaminar tissue (PT) thickness (PTT) were measured from three horizontal and three radial B-scans, respectively. The three horizontal scans were selected from three equidistant locations in the middle third of the affected hemi-ONH (Fig 1F). The radial scans were obtained from the sector of interest: for the superotemporal sector from the 45-, 60-, and 75-degree meridians, and for the inferotemporal sector from the 285-, 300-, and 315-degree meridians (Fig 1I).

The LCD was measured on the three horizontal B-scans. A horizontal reference line was drawn by connecting the two termination points of Bruch’s membrane opening (BMO) in each B-scan image. The LCD was then measured from the reference line to the level of anterior border of the LC at the maximally depressed point and two additional points that were 100 and 200 μm from the maximally depressed point in the temporal direction. The distance was measured on the line perpendicular to the reference line using the manual caliper tool of the Amira software (Amira 5.2.2; Visage Imaging, Berlin, Germany) (Fig 1H).

The PTT was measured on the radial B-scans. A vertical reference line perpendicular to the horizontal reference line was drawn at the center of BMO (Fig 1K). PT was then defined by delineating the area surrounded by the vertical reference line, anterior LC border, border tissue of Elschnig on the temporal side, the horizontal reference line connecting each BMO at the neuroretinal rim, and the internal limiting membrane (Fig 1K). When blood vessels were observed above the PT, they were excluded when delineating the PT. The area of the delineated PT was measured using Image J software (version 1.45s, Wayne Rasband, National Institutes of Health, Bethesda, MD, USA). The average PTT was calculated by dividing the PT area by half of the BMO distance.

The average measurements from the three B-scans were determined as the LCD and PTT for the eye. For NTG patients and healthy control subjects, the LCD and PTT were measured in the matched sector of interest in the same manner. All measurements were performed by two independent observers (E.J.L. and Y.J.C.) who were masked to the clinical information of the subjects. The average of the two measurements (one from each observer) was used for analysis.

To further characterize the PT configuration in NAION, the PTT was measured in the unaffected opposite sectors (e.g., inferotemporal sector in eyes with NAION in the superotemporal sector) in NAION eyes with unilateral sector involvement, and compared with the PTT in the affected sector. The same comparison was also made in NTG eyes in which the disease affected only the hemi-ONH, and in healthy eyes as a control evaluation.

### Data Analysis

Interobserver agreements in measuring the LCD and PTT were assessed by Bland-Altman analysis. Independent comparison among the three groups was performed using the Kruskal-Wallis test with Mann-Whitney *U* post-hoc analysis for continuous variables, and Fisher’s exact test for categorical variables. Paired comparisons of the PTT between affected and unaffected sectors were performed using the Wilcoxon signed-rank test. Statistical analyses were performed using PASW Statistics software (version 20.0.0, SPSS, Chicago, IL, USA). A probability value of *P* < 0.05 was considered statistically significant. Data are presented as mean±standard deviation values except where indicated otherwise.

## Results

This study enrolled 21 NAION patients, comprising 8 women and 13 men. For comparison, 42 age-, ODA-, and RNFL-thickness-matched NTG patients and 42 age- and ODA-matched healthy controls were included. Individual participants’ data are presented in S1 Table. There were no between-group differences in age, gender, VA at diagnosis, prevalence of diabetes mellitus, IOP, central corneal thickness, or ODA (Table 1). The prevalence of hypertension was higher in NAION patients than in NTG patients and normal controls (*P* < 0.001). NTG patients were more myopic (*P =* 0.009) and had a larger axial length (*P* = 0.003) than NAION patients and normal controls (Table 1).

**Table 1:** Patient clinical demographics.

	NAION (n = 21)	Healthy (n = 42)	NTG (n = 42)	*P* value*	Post-hoc^†^
Age at diagnosis, yrs	61.6 ± 10.6	60.0 ± 9.7	60.4 ± 9.8	0.686	
Gender, male/female	13/8	16/26	22/20	0.143	
Visual acuity at diagnosis, logMAR	0.04 ± 0.79	0.04 ± 0.05	0.07 ± 0.13	0.613	
Diabetes mellitus, n (%)	4 (19.0)	4 (9.5)	3 (7.1)	0.407	
Systemic hypertension, n (%)	13 (61.9)	6 (14.3)	10 (23.8)	<0.001	NAION > Healthy = NTG
IOP ^‡^, mmHg	11.7 ± 2.6	11.6 ± 2.6	12.1 ± 2.3	0.602	
Refractive error, diopters	-0.07 ± 1.59	0.12 ± 1.55	-1.52 ± 2.49	0.009	NAION = Healthy > NTG
Central corneal thickness, μm	536.6 ± 30.8	545.8 ± 37.6	554.7 ± 37.7	0.143	
Axial length, mm	23.31 ± 1.05	23.47 ± 1.08	24.17 ± 1.06	0.003	NTG > NAION = Healthy
Visual field MD, dB	-10.80 ± 6.99	-0.25 ± 1.53	-8.93 ± 8.05	<0.001	Healthy > NAION = NTG
Visual field PSD, dB	9.27 ± 3.94	1.63 ± 0.89	8.27 ± 4.77	<0.001	NAION = NTG > Healthy
Optic disc area, mm^2^	2.07 ± 0.26	2.03 ± 0.25	2.07 ± 0.26	0.777	
RNFL thickness of the sector of interest, μm	64.9 ± 21.7	138.5 ± 23.7	66.7 ± 23.0	<0.001	Healthy > NAION = NTG

Data are presented as mean ± standard deviation unless otherwise indicated.

NAION, nonarteritic anterior ischemic optic neuropathy; NTG, normal tension glaucoma; IOP, intraocular pressure; MD, mean deviation; PSD, pattern standard deviation; RNFL, retinal nerve fiber layer.

* Comparison was performed using Kruskal-Wallis test for continuous variables, and Fisher’s exact text for categorical variables.

^†^ Post-hoc analysis was performed using the Mann-Whitney U test.

^‡^ Untreated IOP for NTG patients.

Among 21 NAION eyes, the superior and inferior hemi-ONHs were affected in 11 and 2 patients, respectively. Both hemi-ONHs were affected in 8 eyes, with the superior hemi-ONH being more severely affected in 7 of them. Thus, the superotemporal and inferotemporal sectors were the sectors of interest in 18 and 3 patients, respectively. Accordingly, 36 and 6 NTG patients were selected by matching the superotemporal and inferotemporal RNFL thicknesses, respectively. The RNFL thickness in the matched sector (i.e., the sector of interest) did not differ between NAION and NTG patients (*P* = 0.651, Table 1).

The lower and upper values for the 95% limits of agreement of LCD measurements between the two observers were –22.2 and 15.5 μm, respectively; the corresponding values for measurements of the PTT were –67.3 and 67.2 μm. The LCD was larger in NTG eyes than in healthy control and NAION eyes (*P* = 0.001). The average PTT was largest in healthy subjects followed by NAION and NTG patients (*P* < 0.001) (Table 2).

**Table 2:** Measurements of the LCD and average PTT in the three groups.

	NAION (n = 21)	Healthy (n = 42)	NTG (n = 42)	*P* value*	Post hoc^†^
LCD, μm	390.1 ± 111.8	427.3 ± 94.1	494.2 ± 92.8	0.001	NTG > NAION = Healthy
Average PTT, μm	247.1 ± 75.3	334.7 ± 81.2	178.7 ± 52.1	<0.001	Healthy > NAION > NTG

Data are presented as mean ± standard deviation.

NAION, nonarteritic anterior ischemic optic neuropathy; NTG, normal tension glaucoma; LCD, anterior lamina cribrosa surface depth; PTT, prelaminar tissue thickness.

* Comparison was performed using Kruskal-Wallis test.

^†^ Post-hoc analysis was performed using the Mann-Whitney U test.

**Fig 2:**
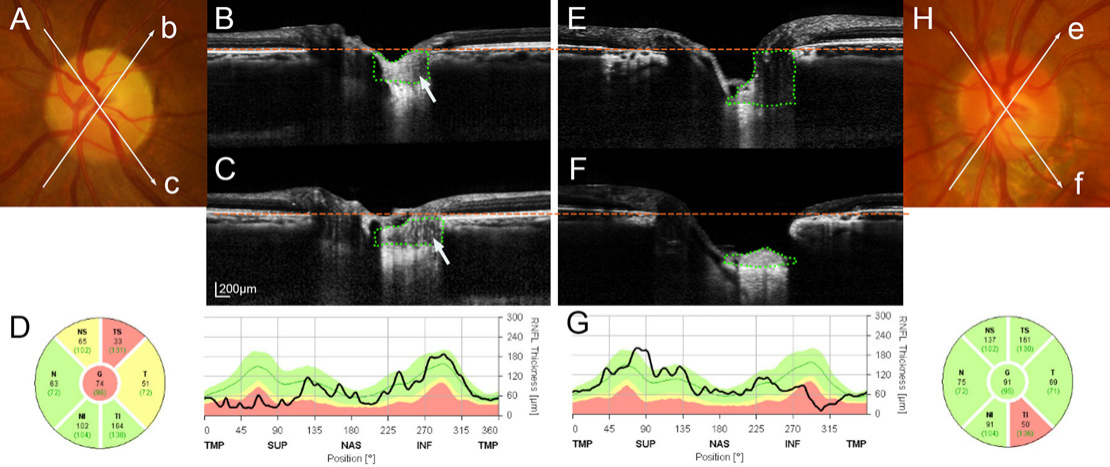
Comparison of the prelaminar tissue (PT) thickness (PTT) between the affected (B, F) and unaffected (C, E) sectors in eyes with nonarteritic anterior ischemic optic neuropathy (NAION) (A-D) and normal-tension glaucoma (NTG) (E-H). *Arrows* in color disc photographs (*b*, *c*; A and *e*, *f*; H) indicate the locations and directions of radial B-scans shown in (B), (C), (E), and (F), respectively. *Orange dashed lines* indicate the level of the Bruch’s membrane opening. G*reen dotted lines* demarcate the area where the PTT was measured. **(A)** Disc photograph of a NAION patient. Note the pallor evident in the superotemporal disc. **(B)** The PT is thick in the affected sector, with the PTT being comparable to that in the unaffected sector shown in the other image (**C**; areas within the *light green dotted lines*). Note that the PT is more homogeneous in the affected sector (B, second left; *arrow*) than in the unaffected sector (C, left; *arrow*). **(D)** RNFL thickness profiles indicate a retinal nerve fiber layer (RNFL) defect in the superotemporal sector. **(E, F)** There is a remarkable difference in the PTT between the unaffected sector (E) and the affected sector (F) (areas within the *light green dotted lines*) in this NTG eye with an untreated intraocular pressure of 14 mmHg. Note that the anterior laminar surface is located deeper than in (B) and (C). **(G)** Abnormally thin RNFL is evident in the inferotemporal sector. **(H)** Note the neuroretinal rim notching in the inferotemporal disc.

The PTT did not differ between the sectors matched to the affected and unaffected sectors in healthy control eyes (*P* = 0.138, Table 3). The PTT did not differ between the affected and unaffected sector in eyes with NAION (*P* = 0.382, Table 3), but it did differ significantly in eyes with NTG (*P* = 0.001, Table 3) (Fig 2).

**Table 3:** Comparison of the PTT between affected and unaffected sectors in the three groups.

	NAION (n = 13)	Healthy (n = 42)	NTG (n = 23)
Affected sector, μm	267.8 ± 65.8	332.2 ± 83.2	196.6 ± 48.9
Unaffected sector, μm	273.7 ± 60.8	319.4 ± 73.6	241.5 ± 91.5
*P* value*	0.382	0.138	0.001

Data are presented as mean ± standard deviation.

For NAION and NTG group, only eyes with hemi-optic nerve head disease were included. For healthy subjects, the comparison was made between the sectors matched for the affected and unaffected sector of NAION and NTG patients.

PTT, prelaminar tissue thickness; NAION, nonarteritic anterior ischemic optic neuropathy; NTG, normal tension glaucoma.

* Comparisons were made using Wilcoxon signed ranks test.

## Discussion

We found that the LCD was larger in NTG patients than in healthy control subjects, while it was comparable between NAION patients and healthy controls. The PT was thinner in both NAION and NTG patients than in healthy controls, and thinner in NTG patients than in NAION patients. To the best of our knowledge, this is the first study to demonstrate the differences in the features of the deep ONH structure between NTG and NAION.

Experimental studies have demonstrated posterior displacement of the LC after IOP elevation in a primate glaucoma model [12, 13]. Conversely, a reduction of the LC depth was demonstrated in glaucoma patients after IOP-lowering treatment [14, 15], and re-displacement of the LC was observed when the IOP was re-elevated [16]. These findings together suggest that the LCD is strongly associated with IOP-related stress. The larger LCD in NTG patients than in healthy control subjects is consistent with the notion that mechanical stress plays an important role in NTG.

It is intriguing that the LCD was larger in the NTG group than in the control group even though the IOP was matched between the groups. There are several possible explanations for this. First, computational modeling studies have demonstrated that mechanical stress applied to the ONH may differ with the geometry of the eyeball even for the same IOP [13, 17–19]. This geometry may differ between NTG and NAION patients so as to amplify the mechanical stress applied to the ONH in NTG. Second, previous studies have suggested that the cerebrospinal fluid pressure may be low in eyes with NTG [20, 21]. In this case the translaminar pressure gradient may be larger in eyes with NTG even for the same IOP, which would induce posterior displacement of the LC. Indeed, our group recently demonstrated that the LCD is larger in eyes with a larger translaminar pressure difference/gradient among healthy subjects [22]. Third, it is also possible that the material properties of the LC differ between NTG and NAION patients (i.e., LC is hypercompliant in NTG) [13]. Finally, the possibility that NTG eyes have an innate deeper LC than healthy subjects and NAION patients cannot be excluded by this cross-sectional study.

While the larger LCD in NTG patients suggests that IOP-induced stress plays an important role, our findings do not necessarily indicate that IOP is the only causative factor associated with the optic nerve damage in NTG. These other factors may induce GON in conjunction with IOP-induced stress or by themselves in some patients.

The PT was thicker in NAION patients than in NTG patients despite their RNFL thicknesses being comparable. This may reflect reactive gliosis, which is known to occur in NAION [23, 24]. Alternatively, the possibility that NAION patients had a thicker innate PT cannot be ruled out. Although this cannot be clearly determined by this cross-sectional study, we consider that a thicker PT in NAION patients would be largely attributable to a higher degree of reactive gliosis for the following reasons. First, the PT was thinner in the unaffected sector than in the matched sector of healthy controls (273.7±60.8 *vs*. 319.4±73.6 μm, *P* = 0.032; Table 3). If NAION patients had an innate thick PT, the PT should be thicker in the unaffected sector than in the matched sector of healthy controls. Second, the PTT did not differ between the superotemporal and inferotemporal sectors in healthy control eyes, which indicates that there is no innate difference in the PTT between these two sectors. In NTG eyes with hemi-ONH involvement, the PT was significantly thinner in the affected sector than in the unaffected sector, indicating that RNFL loss (i.e., axonal degeneration) induces the reduction of the PTT. Given that the parapapillary RNFL is abnormally thin in the affected sector in eyes with NAION, it is also expected that the PT will be thinner in the affected sector than in the unaffected sector in these eyes. However, the PTT did not differ between the affected and unaffected sectors in eyes with NAION. This finding indicates that reactive gliosis may compensate for the loss of neural components in the PT that should have occurred along with the axonal degeneration. Third, the reflectivity of the PT in eyes with NAION differed between the affected and unaffected sectors. The PT in the affected sector appeared more homogeneous and hyperreflective relative to the ipsilateral opposite sector in eyes with NAION (Fig 2). This suggests that the tissue composition had changed in the affected sector.

The reason for cupping being absent in NAION has been unclear, but the results obtained in the current study may give a plausible explanation. The degree of cupping represents the sum of posterior displacement of the anterior LC surface and PT loss [12, 13, 25–27]. Reactive gliosis may play a role in reducing the degree of cupping by compensating for PT loss [23, 24]. Our results suggest that the absence of cupping in NAION is attributable to two factors: (1) a smaller LCD and (2) a thicker PT relative to NTG, which is probably due to reactive gliosis.

The present study was subject to some limitations. First, the observations were performed cross-sectionally, and so it was not possible to assess the true changes in measured parameters relative to their baseline (i.e., unaffected) values. Second, the sample was relatively small and all of the included subjects were Korean. Further studies are needed that involve large numbers of patients from other ethnic groups. Third, previous studies using finite-element modeling have demonstrated that the scleral thickness affects the mechanical stress applied to the ONH [18, 28] and the strains in the LC [17, 28]. These findings suggest that the scleral thickness significantly influences the LCD. However, the scleral thickness was not evaluated in the present study since this is not measurable using EDI SD-OCT due to the invisibility of the posterior (outer) surface of the sclera. Fourth, the LC thickness values were not compared in the present study due to the difficulty of detecting the posterior border of the LC in some NAION and healthy eyes.

In conclusion, the NTG eyes in this study had a larger LCD than the IOP-matched healthy controls. This finding is consistent with IOP-related mechanical stress being associated in NTG. The PT was thicker in NAION eyes than in NTG eyes despite having similar degrees of RNFL loss, suggesting the occurrence of a higher degree of reactive gliosis in the NAION patients. The striking difference in the deep ONH configuration between NTG and NAION may be useful for the differential diagnosis of these two diseases.

## Supporting Information

S1 Table Data of whole participants. (XLSX)
